# Editorial: Plant-Pest Interactions Volume I: Acari and Thrips

**DOI:** 10.3389/fpls.2021.773439

**Published:** 2022-01-12

**Authors:** George Broufas, Felix Ortego, Takeshi Suzuki, Guy Smagghe, Colette Broekgaarden, Isabel Diaz

**Affiliations:** ^1^Department of Agricultural Development, Faculty of Agricultural Sciences and Forestry, Democritus University of Thrace, Komotini, Greece; ^2^Centro de Investigaciones Biologicas Margarita Salas, Consejo Superior de Investigaciones Cientificas, Madrid, Spain; ^3^Graduate School of Bio-Applications and Systems Engineering, Tokyo University of Agriculture and Technology, Fuchu, Japan; ^4^Department of Plants and Crops, Ghent University, Ghent, Belgium; ^5^KeyGene N.V., Wageningen, Netherlands; ^6^Centro de Biotecnología y Genómica de Plantas, Universidad Politécnica de Madrid-Instituto Nacional de Investigación y Tecnología Agraria y Alimentaria, Campus de Montegancedo, Madrid, Spain; ^7^Departamento de Biotecnología-Biología Vegetal, Escuela Técnica Superior de Ingeniería Agronómica, Alimentaria y de Biosistemas, Universidad Politécnica de Madrid, Madrid, Spain

**Keywords:** acari, thrips, plant defense, resistance, multiple interactions

Plants and phytophagous arthropods have shared more than 400 million years of evolution. Consequently, both adversaries have developed physical and chemical barriers to protect against each other. They have not only modified their metabolic pathways and physiology but have also adapted behavior habits, to finally maintain a growth-defense trade-off which ensures their survival. Many publications have appeared in the last years, particularly focusing on the molecular aspects associated with the defense and counter-defense processes involved in the plant-pest relationship (reviewed by Santamaria et al., 2018, 2020; Stahl et al., 2018; Erb and Reymond, 2019).

This Research Topic is addressed in a special issue on plant-pest interactions which has been divided into three volumes based on the pest order. Volume I is dedicated to Acari and thrips, a group of pest species small in size but with a great impact on agricultural production worldwide (Migeon and Dorkeld, 2006–2021; Wu et al., 2018; Sperotto et al., 2019). Although phylogenetically distant, Acari and thrips have two essential characteristics in common: (i) the feeding mode mediated by a stylet which facilitates sucking from mesophyll or epidermal cells (Kindt et al., 2003; Bensoussan et al., 2016), and (ii) the ability to develop resistance to pesticides used to control them (van Leeuwen and Dermauw, 2016; Steenbergen et al., 2018). The six articles included in Volume I add novel insights at the physiological and molecular levels on plant-Acari/thrip interactions as well as new experimental procedures to work with these pests.

Among thrips, *Frankliniella occidentalis* Pergande (Thysanoptera: Thripidae) is the most intensively studied species because it causes direct damage by feeding on a wide range of crops and acts as an important transmitter of viral diseases (Rotenberg and Whitfield, 2018; Rotenberg et al., 2020). It is the most efficient vector of the tomato spotted wilt virus (TSWV), which is ranked in the top 10 most important plant viruses worldwide. Previous data had reported that as a consequence of the TSWV transmission, indirect or plant-mediated effects were produced on the vector-plant interaction, by altering plant physiology and benefiting vector fitness (Ogada et al., 2013; Wu et al., 2019). In this research context, Nachappa et al. investigated the tomato-mediated molecular mechanisms underlying the TSWV-*F. occidentalis* relationship. Microarray assays performed in tomato, mock or systemically-infected with TSWV and subsequently infested with or without thrips, revealed that TSWV is the main driver of the plant responses. Either TSWV alone or in combination with thrips suppressed genes involved in host primary metabolism, altered the expression of genes associated with hormone defense signaling, and upregulated genes involved in protein metabolism. Consequently, the total free amino acid content was increased and plants became more suitable hosts for thrips. So, the dual attack compromised plant health and defenses.

Spider mites (Acari: Tetranychidae) are the most economically important group of phytophagous mites leading to serious agricultural losses all over the world. Defenses developed by plants against spider mites have been widely investigated in model and crop species in the last decades. Most studies have compared transcriptomic, proteomic, and metabolomic data derived from infested susceptible and resistant accessions/cultivars to identify key genes/proteins/molecules with altered abundance via spider mite infestation (Zhurov et al., 2014; Hoseinzadeh et al., 2020; Zhang et al., 2020). The article published by Weinblum et al. combines transcriptome and metabolome analyses to obtain a comprehensive insight into the defense responses of domesticated tomato cultivars against the polyphagous spider mite *Tetranychus urticae*. Results revealed changes in genes associated with primary and secondary metabolism, including hormones and volatiles. The major significant differences dealt with monoterpenoid and phenylpropanoid volatiles induced in infested resistant cultivars, which were consistent with transcriptomic data. Olfactory choice bioassays with *Phytoseiulus persimilis*, a predator of mites, showed exclusive attraction for infested resistant tomatoes which corroborated the defense role of these metabolites. Other aspects of the plant-mite relationship were studied by Jiwuba et al. who evaluated 60 cassava genotypes across different environments on the resistance of the cassava green mite *Mononychellus tanajoa* (Tetranychidae), and their effects on cassava yields in Nigeria. The end goal was to determine their adaptability and find genotypes that could be potentially recommended for cultivation. They identified four cassava genotypes that were more stable and resistant to *M. tanjana*, which combined with useful agronomic traits could be selected as preferred cassava genotypes to be adopted by farmers. This is practical work to provide superior cassava plants, considered as an essential staple food and animal feed in tropical and sub-tropical Africa.

In an article reported by Ghazy et al. a new method is described that uses a sheet-like structure to mimic plant leaves for delivering experimental solutions to stylet-feeding arthropods. The flexibility of the method was tested with three acarine and one aphid species and allowed large-scale screens of active ingredients and/or pesticides for pest control.

A systematic review by Garcia et al. presented a meta-analysis to evaluate the effects of induced plant defenses produced upon pest feeding on plant fitness and surrogate parameters. The information on defense-growth trade-offs is of great help to the scientific community for the design of pest management strategies and reducing costs.

Finally, a perspective article by Arnaiz et al. compiles the current, although still limited knowledge, on nitric oxide (NO), either as a signal molecule, a metabolic intermediate, or a toxic oxidative product in the generation of plant defenses against insects and plant feeding mites, and particularly in *T. urticae*, as well as the contribution of other molecules associated with NO metabolic pathways.

The information reported in Volume I on plant-pest interaction has enlarged the knowledge on the plant-Acari/thrip interplay, and has added new experimental methods and novel perspectives, but further research is required to obtain full understanding-driven sustainable control against a diverse range of pest mites and thrips.

## Author Contributions

ID wrote the editorial with contributions from all GB, FO, TS, GS, and CB. All authors acted as co-editors of this special issue and approved the submitted version.

## Conflict of Interest

CB was employed by company KeyGene. The remaining authors declare that the research was conducted in the absence of any commercial or financial relationships that could be construed as a potential conflict of interest.

## Publisher's Note

All claims expressed in this article are solely those of the authors and do not necessarily represent those of their affiliated organizations, or those of the publisher, the editors and the reviewers. Any product that may be evaluated in this article, or claim that may be made by its manufacturer, is not guaranteed or endorsed by the publisher.
